# Interdisciplinary communication and collaboration as key to improved nutritional care of malnourished older adults across health‐care settings – A qualitative study

**DOI:** 10.1111/hex.13075

**Published:** 2020-06-11

**Authors:** Marije H. Verwijs, Saskia Puijk‐Hekman, Ellen van der Heijden, Emmelyne Vasse, Lisette C. P. G. M. de Groot, Marian A. E. de van der Schueren

**Affiliations:** ^1^ Department of Nutrition and Health HAN University of Applied Sciences Nijmegen The Netherlands; ^2^ Dutch Malnutrition Steering Group Amsterdam The Netherlands; ^3^ Division of Human Nutrition and Health Wageningen University Wageningen The Netherlands

**Keywords:** continuity of patient care, elderly, interdisciplinary communication, malnutrition, transitional care

## Abstract

**Background:**

Malnutrition is a risk factor for impaired functionality and independence. For optimal treatment of malnourished older adults (OA), close collaboration and communication between all stakeholders involved (OA, their caregivers and health‐care and welfare professionals) is important. This qualitative study assesses current collaboration and communication in nutritional care over the continuum of health‐care settings and provides recommendations for improvement.

**Methods:**

Eleven structured focus group interviews and five individual interviews took place in three regions across the Netherlands from November 2017 until February 2018, including OA, caregivers and health‐care and welfare professionals. Various aspects of collaboration and communication between all stakeholders were discussed. Interviews were transcribed and analysed using a thematic approach.

**Results:**

Six main themes emerged: causes of malnutrition, knowledge and awareness, recognition and diagnosis of malnutrition, communication, accountability and food preparation and supply. Physical and social aspects were recognized as important risk factors for malnutrition. Knowledge and awareness regarding malnutrition were acknowledged as being insufficient among all involved. This may impair timely recognition and diagnosis. Responsibility for nutritional care and its communication to other disciplines are low. Food preparation and supply in hospitals, rehabilitation centres and home care are below expected standards.

**Conclusion:**

Many stakeholders are involved in nutritional care of OA, and lack of communication and collaboration hinders continuity of nutritional care over health‐care settings. Lack of knowledge is an important risk factor. Establishing one coordinator of nutritional care is suggested to improve collaboration and communication across health‐care settings.

## 1 INTRODUCTION

Currently, 19% of the total Dutch population is aged 65 years and older, and this percentage is expected to rise to 26% in 2040.^1^, ^2^ Longevity is not by definition associated with healthy ageing. At older age, the majority of older adults (OA) suffer from multimorbidity,^3^ leading to increased health‐care needs. Yet, Dutch health‐care policy is aimed at encouraging OA to live at home as long as possible, assisted by family and health‐care professionals, when necessary.^4^ Herein, they are expected to coordinate their own health, relying on (remaining) physical function and self‐management as much as possible.^5^ Maintaining a good nutritional status plays an important role in the physical and mental well‐being of OA and may be crucial to living at home as long as possible.

Nutritional risk factors seem highly prevalent among Dutch community‐dwelling OA (CDOA). A recent study based on SCREEN II found that over 80% (n = 2470) of Dutch CDOA has more than one nutritional risk factor,^6^ such as eating alone, difficulties doing groceries, poor appetite and mobility limitations. These nutritional risk factors are common in older age and can lead to a decreased nutritional status and eventually malnutrition.^7^

Malnutrition is characterized by loss of body weight and muscle mass, resulting in immune dysfunction and increased risk of falls, slower recovery and increased risk of complications in disease and after surgery.^8^ The prevalence of malnutrition is reported to range from 11% to 35% in Dutch CDOA,^9^ whereby prevalence rates increase with age.^6^, ^9^ The most vulnerable patients at nutritional risk may be the ones who are in transition of care, for example from hospital or rehabilitation centre to home.^10^ Furthermore, malnutrition risk factors are more likely to occur in persons with higher care complexity^11^ as malnutrition is often co‐existent with other geriatric symptoms such as depression or frailty.^12^ Thus, optimal nutritional care and malnutrition screening and treatment should be an interplay between different health‐care and welfare professionals (professionals), centred around the patients and their caregiver(s). However, general practice proves the opposite.^13^ Ideally, nutritional care and malnutrition prevention and treatment should be a continuum across health‐care settings.^14^, ^15^ So far, little research is available investigating nutritional care collaboration and communication between OA themselves, their caregivers and professionals across the continuum of health care. This qualitative study was to investigate how nutritional care collaboration and communication is organized in the Netherlands, by investigating possible barriers, facilitators, stakeholders’ experiences (OA, caregivers and professionals), wishes and needs in order to optimize collaboration and communication in nutritional care.

## 2 METHODS

### 2.1 Design

A qualitative design was used to investigate current practice and to assess possible barriers and facilitators with regard to collaboration and communication in nutritional care in Dutch OA across health‐care settings. Focus group interviews with Dutch OA, their caregivers (social network; ie relatives, friends, neighbours) and professionals (ie GPs, nurses, dietitians, social workers, cooks) were held to collect all necessary information and to discover possible niches.

Between November 2017 and February 2018, focus group interviews were held in three different regions across the Netherlands: Gorinchem, Sneek and Nijmegen. These include both rural and urban regions. They were held on familiar locations, nearby the residence or working area of the respondents. SPH, a trained moderator, conducted the focus group interviews, supported by local project leaders, who made notes about non‐verbal communication. Respondents were asked to share their experiences with nutritional care across health‐care settings and were encouraged to be as candid about their thoughts as possible. Focus group interviews lasted 1.5‐2 hours and were audio‐taped.

### 2.2 Study population and recruitment

Community‐dwelling OA and caregivers were recruited for participation through personal approach in community centres or with the help of OA’s associations and personal contacts. No specific inclusion or exclusion criteria were applied with regard to the respondents’ usual nutritional habits or risk of malnutrition. Additionally, to study communication across health‐care settings, OA who stayed at a rehabilitation centre (after a hip fracture, in transition to home) were invited by local project leaders. Local project leaders approached professionals employed at hospitals, rehabilitation centres and in home care within their network, and through snowball sampling. Four focus group interviews with adults aged 65 years or older (n = 18; Table 1) and caregivers (n = 5; Table 2) took place.

**Table 1:** Baseline characteristics of respondents to focus groups and individual interviews: older adults

	Total	Gorinchem	Nijmegen	Sneek
n	21	9	7	5
Mean age (y)	71.6	72.4	71.7	70.8
Age range (y)	58‐88	65‐86	58‐88	67‐77
Sex				
Female	14	8	3	4
Male	5	1	4	1
Education				
Primary education	2	‐	2	‐
Lower vocational/advanced secondary education	10	6	2	2
Intermediate vocational/highersecondary education	2	2	‐	‐
Higher vocational education/university	6	‐	3	3
Unknown	1	1	‐	‐
Marital status				
Single	3	1	1	1
Married/living together	10	3	5	2
Married/living apart	1	1	‐	‐
Divorced	2	2	‐	‐
Widow(er)	5	2	1	2
Other	‐	‐	‐	‐
Help at home				
None	12	3	4	5
Caregiver (children, family, friends, neighbours)	3	1	2	‐
Home care	4	2	2	‐
Household help	6	5	1	‐
Meal service	‐	‐	‐	‐
Other	‐	‐	‐	‐
Nutritional advice by				
None	8	4	2	2
GP	6	2	4	‐
GP’s nurse practitioner	4	2	1	1
Medical specialist	2	1	1	‐
Dietitian	8	3	3	2
Physiotherapist	2	1	1	‐
Occupational therapist	‐	‐	‐	‐
Speech therapist	‐	‐	‐	‐
Pharmacist	2	‐	2	‐
Dentist	2	‐	2	‐
Other	‐	‐	‐	‐

**Table 2:** Baseline characteristics of respondents to focus groups and individual interviews: caregivers

	Total	Gorinchem	Nijmegen	Sneek
n	5	2	1	2
Mean age (y)	66.5	49.5	89	61
Age range (y)	32‐89	32‐67	89	51‐71
Sex				
Female	4	1	1	2
Male	1	1	‐	‐
Education				
Primary education	‐	‐	‐	‐
Lower vocational/advanced secondary education	1	1	‐	‐
Intermediate vocational/higher secondary education	‐	‐	‐	‐
Higher vocational education/university	4	1	1	2
Unknown	‐	‐	‐	‐
Marital status				
Single	1	‐	1	‐
Married/living together	4	1	1	2
Married/living apart	‐	‐	‐	‐
Divorced	‐	‐	‐	‐
Widow(er)	‐	‐	‐	‐
Other	‐	‐	‐	‐
Help at home				
None	3	2	‐	1
Caregiver (children, family, friends, neighbours)	‐	‐	‐	‐
Home care	1	‐	‐	1
Household help	2	‐	1	1
Meal service	‐	‐	‐	‐
Other	‐	‐	‐	‐
Nutritional advice by				
None	3	2	1	‐
GP	‐	‐	‐	‐
GP’s nurse practitioner	‐	‐	‐	‐
Medical specialist	‐	‐	‐	‐
Dietitian	1	‐	‐	1
Physiotherapist	‐	‐	‐	‐
Occupational therapist	‐	‐	‐	‐
Speech therapist	‐	‐	‐	‐
Pharmacist	‐	‐	‐	‐
Dentist	‐	‐	‐	‐
Other	‐	‐	‐	‐

After the focus group interview with OA and caregivers living in Nijmegen, it appeared that a few respondents did not feel safe enough to share all their experiences during the focus group. One respondent was not able to be present during the focus group. In order to complete OA’s experiences, supplementary individual interviews were held with four of the respondents of that particular focus group.

In addition, one couple from Moroccan origin was recruited from the Nijmegen area. Both respondents were not fluent in Dutch; therefore, an interpreter was present during the interview. The interpreter was a social worker who supported the couple. Additionally, the couple’s daughter was also present during the interview. The couple indicated that they did not want the interview to be audio‐taped. Therefore, notes were made and summarized, and the couple signed this summary of the interview.

The individual interviews with the couple from Moroccan origin and the four respondents from Nijmegen lasted 30 minutes to 1 hour and were also audio‐taped. Baseline data of the interviews are added to Table 1.

Seven focus group interviews with professionals took place, including 41 care professionals in total. The professionals who participated in the study included different disciplines in health care and welfare (Table 3).

**Table 3:** Baseline characteristics of respondents to focus groups: health‐care and welfare professionals

	Total	Gorinchem	Nijmegen	Sneek
n	41	16	12	13
Mean age (y)	44.3	44.9	39.9	47.5
Age range (y)	21‐63	21‐61	21‐62	23‐63
Sex				
Female	34	12	10	12
Male	7	4	2	1
Education				
Primary education	‐	‐	‐	‐
Lower vocational/advanced secondary education	7	4	‐	3
Intermediate vocational/higher secondary education	1	‐	‐	1
Higher vocational education/university	33	12	12	9
Employed at				
Hospital	10	2	5	3
Rehabilitation centre	10	7	‐	3
Home care	15	4	7	4
Combination	6	3	‐	3
Profession				
GP	1	1	‐	‐
GP’s nurse practitioner	2	‐	‐	2
Dietitian	3	1	1	1
Physiotherapist	3	1	1	1
Speech therapist	4	2	1	1
Social worker	5	‐	4	1
Nurse	10	4	3	3
Geriatrician	1	1	‐	‐
Psychologist	1	1	‐	‐
Occupational therapist	2	1	‐	1
Cook	1	‐	‐	1
Nutrition assistant	1	‐	‐	1
Business manager local hospital	1	‐	1	‐
Health broker	1	‐	1	‐
Care coordinator	3	2	‐	1
Sports coach	1	1	‐	‐

All interviews were transcribed verbatim by an external party. Respondents’ names were replaced by pseudonyms to ensure anonymity and confidentiality.

### 2.3 Focus group interviews

Discussion guides were developed based on Evers^16^ to ensure all key‐concept areas were discussed. The topics of the discussion guide consisted of two main topics including 12 subtopics, presented in Table 4.

**Table 4:** Construction of the discussion guides

Main topic	Subtopic
Identifying barriers and facilitators in nutritional care across health‐care settings	Problem detection
	Application of existing guidelines
	Expectations of clients/health‐care professionals
	Personal care plans
	Continuity in care between health‐care settings
	Transfer report
	Coordination of care and welfare
	Case management
	Inventory and involvement of stakeholders
	Advanced care planning
Inventory of experiences, wishes and needs of older adults, caregivers and health‐care professionals	Facilitators
	Barriers

### 2.4 Ethical issues

The study was judged by the HAN Ethical Advisory Board, and they advised that no further ethical approval was necessary, as ‘The study does not fall within the remit of the Medical Research Involving Human Subjects Act (WMO)’. All respondents received detailed information about the aim of the study and the content of the focus group interviews in advance. Furthermore, respondents were requested to sign an informed consent form and they were aware of the right to withdraw at any time.

### 2.5 Data analysis

The interviews were analysed using a thematic approach.^17^ Prior to data analysis, two authors (SPH and MHV) listened to the recorded interviews separately. They went through the data and searched for meanings and patterns to make an initial list of recurring topics, after which the formal coding process started. Both authors assigned open codes to pieces of text that had the same underlying meaning, using Atlas.ti version 8.2 (Atlas.ti Scientific Software Development GmbH). Codes were discussed and discrepancies were managed by consensus. In case discrepancies arose, the codebook was revised and previous transcripts were recoded. Codes were grouped into categories and categories were then classified into themes. In case topics occurred that could not be grouped under the topics that were identified initially, extra themes were added in order to include possible niches in the analysis.

## 3 RESULTS

After nine of the 11 focus group interviews, saturation was considered to be achieved. No extra codes nor themes arose during the analysis of the remaining focus group interviews and personal interviews. The following five themes were identified: (i) Causes of malnutrition; (ii) Knowledge and awareness; (iii) Recognition and diagnosis of malnutrition; (iv) Communication; (v) Accountability. While coding the interviews, another theme arose naturally before the formal coding process started: (vi) Food preparation and food supply; within each focus group interview, something was said about food supply and food preparation during and after admission to hospital or rehabilitation centre. Barriers and facilitators with regard to collaboration and communication in nutritional care across the continuum of health‐care settings were discussed.

### 3.1 Causes of malnutrition

#### 3.1.1 Physical decline

Physical decline was recognized as an important cause of malnutrition. Also loss of appetite and taste, the ability to take care of oneself, dementia and cognitive decline, side‐effects of medication and dysphagia were frequently mentioned. These aspects were said to cause shame and fear, such as the fear of falling. This, in turn, often results in a vicious circle in which OA continued to deteriorate physically.

#### 3.1.2 Social aspects

Also social aspects were mentioned as possible causes of malnutrition. A small or shrinking network and/or loneliness were said to have a major influence on appetite and the positive experience of eating and drinking. Additionally, OA come from a generation that is reluctant to ask for help from their social network or from professionals. Another important social aspect mentioned is the importance of proper meals in relation to what they cost.

Various opportunities were mentioned in response to the social aspects described above. An important opportunity could lie within social cohesion of neighbourhoods. Social–cultural and medical facilities could improve social networks around OA and could be an easily accessible way for OA to do groceries and prepare and consume meals together. Quotes of Paragraph 3.1 are depicted in Table 5.

**Table 5:** Quotes Paragraph 3.1 ‘Causes of malnutrition’

*Quote 1:*
Physiotherapist: ‘What I encounter in the home situation in terms of nutrition and medication problems and things you see when you come in people's homes, then, it often starts with less appetite, not feeling well, postponing meal moments, not finishing meals or just forgetting about meals. They find it hard to take care of themselves, and lose their self‐sufficiency’
*Quote 2:*
Nutrition assistant: ‘No, but I think that's also the fact that, isolation of the older adults. You have to sit alone at the table, that's what a lot of people refuse to do […] and if no one is around who says “come on, let's sit at the table together”, that's the thing, then they'll eat a lot less’
*Quote 3:*
Cook: ‘[…] and besides that, there's the financial problem, we all want that everyone gets a proper meal, which is rich in all nutrients, freshly cooked. But often, the family checks the price of such meals […]’
*Quote 4:*
OA: ‘[…] there should be a medical‐social facility, consisting of a place where one can eat, a place where one can get a blood test, a place where a physiotherapist or podiatrist or whatever, where they have their small consulting rooms, where older adults can be cared for’
*Quote 5:*
OA1: ‘It also depends on what the older adult is still capable of themselves’ OA2: ‘And how much family they still have’ OA1: ‘And how much help someone has or if there is a caregiver or professional help involved’

### 3.2 Knowledge and awareness

All focus group interviews revealed that there is a lack of knowledge and awareness towards malnutrition, its causes and consequences (Table 6). Also, the importance of nutrition on health and well‐being was under‐recognized.

**Table 6:** Quotes Paragraph 3.2 ‘Knowledge and Awareness’

*Quote 6:*
Care coordinator: ‘[…] many people don't know the importance of nutrition. And I really think we should do something about it, I also notice it when I am on the phone with people, when I say nutrition is your medicine and you really need to keep eating healthy, “Oh really? Oh malnutrition?” everyone reacts so surprised’
*Quote 7:*
Caregiver (CG): ‘[…] due to the nutritional supplements, he regained some weight and strength, but from the health care professionals involved, no, that is a very blind spot for doctors, nurses, nutrition and weight. […] apparently it is not a matter that really has their attention’

Respondents suggested that the solution may lie in giving more information: OA should be well informed about the causes, consequences and solutions to malnutrition, for example through newspapers or magazines or via the television. Caregivers and health‐care professionals wanted to be educated how they can detect signs of malnutrition and undertake adequate interventions.

### 3.3 Recognition and diagnosis of malnutrition

As stated before, lack of knowledge and awareness was recognized a factor withholding stakeholders from taking preventive measures or starting adequate interventions. Furthermore, professionals do not always know which professional should be consulted if they recognize signs of malnutrition. In order to consult the right professional, it is required to assess the cause of malnutrition. For example, malnutrition caused by loneliness needs another approach than malnutrition caused by dysphagia.

Another factor that was mentioned explaining the missed diagnosis of malnutrition is the fact that a significant group of frail OA remains beneath the radar, even when health problems arise. This group of OA tends to avoid any involvement of professionals or others because they believe they are able to take care of themselves or do not want to be a burden to others, herewith making monitoring, diagnosing and treatment unable. Quotes on recognition and diagnosis of malnutrition can be found in Table 7.

**Table 7:** Quotes Paragraph 3.3 ‘Recognition and diagnosis of malnutrition’

*Quote 8:*
Social worker: ‘[…] I think that there is a large group that remains beneath the radar, and I think you should focus on that group’
*Quote 9:*
GP: ‘They say: “It's all going well doctor”, but when they are admitted to hospital, they can't keep saying that, but prior to the admission, that's the answer you get’.
Sports coach: ‘I can do it by myself easily’

### 3.4 Communication

During all interviews, communication was stated to be an important factor in the treatment and prevention of malnutrition among OA. Several barriers and facilitators were mentioned. Three categories can be distinguished within this theme, which are interrelated: communication between health‐care professionals, communication towards OA and caregivers and communication with regard to the transfer report to another health‐care setting.

#### 3.4.1 Between professionals

Communication with other health‐care professionals is essential once one of the health‐care professionals has identified a (nutritional) problem. In this way, no duplication of work arises and the right health‐care professional can be involved. Unfortunately, the interviews revealed that this is not happening yet. For example, results of nutritional screening (by nurses) are not always shared with the doctor or dietitian and no action is taken. Furthermore, it was acknowledged that lack of communication between professionals resulted in cases where patients needed to tell their personal experiences multiple times, or important information remained unknown. It was suggested that these problems could be resolved easily, by interconnecting existing electronic care systems in which professionals can share their findings. However, this is thought to be problematic, given the European General Data Protection Regulation (GDPR).

#### 3.4.2 Towards OA and caregivers

Within the health‐care system of the Netherlands, self‐management has high priority. Several aspects with regard to the communication towards OA and caregivers were mentioned. Respondents stated that information about their health should be given directly to OA, and OA should be involved in the communication about their own health. Also, health advice and contact with health‐care professionals and social–medical facilities should be easily accessible. When including OA in the communication, this is thought to result in equality between OA and their health‐care professionals, and this could create a relationship of trust, which could make it easier for the OA to share possible health problems. On the other hand, respondents feared that not every OA is able to self‐manage his/her own life and health, for example due to vulnerability or not having the knowledge to do so.

#### 3.4.3 Transfer report

Within the health‐care system in the Netherlands, a transfer report is issued when a patient is discharged from an institution to home. This report includes information about a patient’s disease, recovery and medication use. Unfortunately, in some cases transfer reports are not issued at all, or issued only days after discharge, causing a delay in information. In some cases, the transfer report is sent to the GP but not to other relevant professionals, or patients receive the transfer report themselves, again leading to a delay in provision of information. In addition, several respondents identified that information about a patient’s nutritional status and the involvement of a dietitian or the use of oral nutritional supplement is often not mentioned in such reports. A paper/digital ‘nutritional passport’, or even an overall ‘health‐care passport’, has been suggested as a means of communication. In this case, someone should be made responsible for keeping such a document up to date. This topic relates to the theme discussed in the next chapter. Quotes of Paragraph 3.4 are depicted in Table 8.

**Table 8:** Quotes Paragraph 3.4 ‘Communication’

*Paragraph 3.4.1* Communication between health‐care and welfare professionals	*Quote 10:*
	Interviewer: ‘[…] but actually you would want to prevent that a client needs to tell the same story over and over again, which is actually already known in primary care, right?’Nutrition assistant: ‘In case of some older adults indeed. Yes that would be nice’Interviewer: ‘Yes, that would save a lot of talking but might also give the client the feeling of “well, my situation is already known” and that will give them some more confidence maybe’Nurse: ‘Yes, that as well, and it saves us a day or two of identifying what someone can or cannot do, a decline in exercise or nutrition’Later on in the same interview:Nutrition assistant: ‘[…] I am not going to check the whole nursing record, then I’ll just ask the patient’
*Paragraph 3.4.2* Towards older adults and caregivers	*Quote 11:*
	CG: ‘With regard to older adults, one often speaks about older adults but rarely with older adults. That's the thing throughout the entire civil world and also in the field service and so on, they think they know what's good for us and for some older adults, but not all older adults are already senile and that is often forgotten. So participation is a nice term but if you indeed want to join in the discussion at a given moment, then they are completely upset because then they no longer know what to do’
	*Quote 12:*
	GP’s nurse practitioner: ‘the moment you see a patient for the first time, there are sometimes things that you think, what would that be like and then if you then have a follow‐up appointment with that patient you will see that you get a bond and that you get more information’
*Paragraph 3.4.3* Transfer report	*Quote 13:*
	GP: ‘[…] sometimes you receive the report four days after a vulnerable older adult returns home, you would say that four days would be acceptable, but a lot can happen. In my opinion, I should receive that report before the patient is at home’
	*Quote 14:*
	Nurse: ‘I also think that with regard to the transfer from the hospital, in our practice nutrition isn't included in the transfer report. There is no specific focus on that’

### 3.5 Accountability

During all focus group interviews, the GPs and their nurse practitioners were often referred to as being key figures in the (nutritional) care of OA: they are supposed to have all the information about a client, to know when a client is admitted or discharged from the hospital, when a client experiences (health) problems at home and which health‐care professionals are involved in their clients’ care (Table 9).

**Table 9:** Quotes Paragraph 3.5 ‘Accountability

*Quote 15:*
Occupational therapist: ‘not to put the responsibility on the shoulders of the GP, but he has the overall picture’
Respondent: ‘That's what you're hoping for, he actually remains the gatekeeper for everything’
*Quote 16:*
Occupational therapist: ‘Yes, we also kinda always come back to the GP’s nurse practitioner, because he/she also has the function, they are busy with working that out, there is already a part of where our question simply can be added and he/she can bring all the information together and pass it on to other health care professionals if needed, and can also provide feedback to the GP, and the GP will of course know that immediately. This can all be done in one system’
*Quote 17:*
CG: ‘In my opinion, the nurse from the home care organization can very well evaluate the nutritional intake of the older adults’
*Quote 18:*
Nurse: ‘What I do find difficult in home care, is that you cannot force anything. So you are always dependent on the clients’ goodwill. Also, you're not always there, so you can only stimulate, motivate and monitor whether someone is not losing weight or becoming dehydrated or whatever, and that is already a big step, isn't it?’
*Quote 19:*
Nurse: ‘[…] and that's when it gets debatable, that health insurances say that nutritional care is not part of personal care, so you almost immediately get financial problems. In our organization we decided that we will do it, we are not going to literally feed someone, but we will heat meals and serve breakfast and so on’.
*Quote 20*:
Nutrition assistant: ‘[…] many health care professionals are involved with nutritional care and when talking about a nutritional passport, you would want someone to be responsible, who manages it and that's the question, who is going to manage, to all those things, we all do different tasks and I am wondering who would be responsible for this. Would this be the dietitian who can set the lines and how someone's diet should look like and engage everyone, or should this be a medical specialist, someone's care taker, it can be anyone, but someone should monitor, should it be a GP? […]’
*Quote 21:*
CG: ‘[…] you could also make sure that older adults themselves keep all adequate data and make clear they are in charge, so you could also leave that to older adults themselves, I don't know..’
*Quote 22*:
OA1: ‘It remains true that the whole principle of self‐sufficiency of your health care and that it is, because if you cannot do that, then you won't get it either. That is the essence of the problem, if you cannot do that, there will be gaps’OA2: ‘That is the nasty thing about that whole political load behind that, that we've been called back since two years, well we, but not me, the self‐management in health care and those people have been completely pampered since World War II, a great deal has literally been pre‐chewed, even with regard to meals, with the television and all other comforts and then from 2014 onwards they are expected to self‐direct everything […]’
*Quote 23*:
Interviewer: ‘What I hear is that you have to be very empowered to be able to state things and I don't know if you think, with regard to the other older adults at the rehabilitation unit, would everyone be empowered enough?’OA: ‘No, there are people who cannot do that, they are too sad, or unstable’

The role of home care nurses also appears to be of great importance. They are expected to be able to evaluate nutritional status easily, since they visit their clients at home. However, with regard to taking action, they are limited in time to spend with their client. Formally, they can only signal and monitor nutritional status. Preparing and assisting with meals no longer falls under their duties. However, it depends on the individual employee or home care organization whether they will still assist in serving meals.

A ‘personal (nutrition/health‐care) passport’ was suggested as being one of the solutions to a better communication between health‐care professionals. As to whom should be made responsible for keeping it up to date, the GP, nurse practitioner and/or home care nurses were often being mentioned. Also health‐care mediators and dietitians, and even caregivers were suggested as being appropriate for the job. However, a great deal of responsibility already rests on the caregivers’ shoulders and this may be too much for them to handle. Lastly, it was suggested that OA themselves could be made responsible. This is in line with the previously mentioned policy of self‐management. However, stakeholders doubted whether this will be feasible and effective: the current generation of OA is not used to this responsibility and they might be too vulnerable, too shy to ask for help, or even too dependent on the idea that they will be taken care of by health‐care professionals or caregivers.

### 3.6 Food preparation and supply

Much discussed topics are the quality of the food supply in hospitals and rehabilitation centres, the existence, quality and familiarity of meal services and ready‐made meals, use and quality of oral nutritional supplements, and the enjoyment of food at an older age. Together, these topics form the overall theme *food preparation and supply*.

Several OA who (had) stayed in a hospital and/or rehabilitation centre stated that the quality of the food served was poor. They lacked variation in meals or meal components and were unable to continue their own diet (Table 10). With regard to the quality and familiarity of meal services and ready‐made meals, OA are aware of their existence, but believed that those meals are not healthy (eg too much salt or too big a portion size). With regard to the use of oral nutritional supplements, professionals often noticed that OA do not like the taste or even forget about taking them. Consequently, they come across large amounts stocked in cupboards and refrigerators. All stakeholders indicate that OA often forget about or skip meals, because of a decrease in the enjoyment of food. This could be a result of changes in their social environment, or a decline in smell or taste. Tailor‐made care was mentioned as a possible solution to these problems, taking the ability to taste and smell, taste preferences and social aspects into account.

**Table 10:** Quotes Paragraph 3.6 ‘Food preparation and supply’

*Quote 24:*
OA: ‘And here I notice that the vegetables and the food repeats very often. I am here for two weeks now, and we already had red cabbage three times now with stew, I think that's a lot in only two weeks’ time’
*Quote 25:*
OA: ‘[…] at home you just eat more vegetables, with a salad and chicory and endive’.
*Quote 26:*
OA1: ‘Ready‐made meals, I don't know if you would like that, but you only have to put those in a microwave’OA2: ‘Yes but those meals are such big portions, I only eat a small amount per day and when I look at those ready‐made meals I think: hello, I can have dinner twice!’OA3: ‘And they contain a lot of salt’
*Quote 27:*
GP: ‘What I also encounter frequently is that a patient is being viewed very clinical, the patient loses weight, so medical nutrition is being prescribed. And when you visit them at home you see a refrigerator full of bottles of medical nutrition that people just can't consume, or you see that someone cannot eat regular nutrition anymore’

### 3.7 Interview couple from Moroccan origin

The interview with the couple from Moroccan origin revealed that they expressed themselves differently from people with a Dutch origin. They indicated that they dealt with illness differently and that care is mainly provided by their family members and caregivers. Consequently, they rely less on professionals. When in touch with health‐care professionals, language barriers have been a major problem. Because of the language barrier, the couple experienced that they were not always involved in decisions about their own health care. Furthermore, they indicated that during hospital admission they were not sure whether the food was hallal. They also believed that discharge from hospital to home was too soon, the respondent was not fully recovered. Little had been arranged by the hospital, and home care was not available. At discharge, the respondent received the transfer report and it was the respondents’ responsibility to make sure the report was delivered to their GP.

### 3.8 Suggestions

The respondents made different suggestions to optimize collaboration and communication across the continuum of health care. Table 11 includes these suggestions, summarized per theme.

**Table 11:** Suggestions for collaboration and communication in nutritional care across the continuum of health‐care settings

Theme	Suggestions
Causes of malnutrition	Social cohesion: social–cultural and medical facilities to improve social networks of older adults
Knowledge and awareness	Inform older adults about the causes, consequences and solutions of malnutrition, through local newspapers, supermarkets’ magazines and television
	Inform caregivers and health‐care professionals about detection and treatment of malnutrition
Diagnosis of malnutrition	Communicate (nutritional) problems with other health‐care professionals by using current communication systems
Communication	Include older adults in communication about their own health
	Include nutritional information in transfer reports
Accountability	One single health‐care professional should be appointed to coordinate nutritional care, consulting the right health‐care or welfare professional and monitoring health goals in co‐operation with the older adult

## 4 DISCUSSION

To our knowledge, this is one of the first qualitative studies into barriers, facilitators and needs for collaboration and communication in nutritional care across health‐care settings. The results of this study show that many stakeholders are involved in nutritional care, but communication and collaboration between stakeholders often fall short and expectations cannot be met. Prerequisites for interdisciplinary communication and collaboration in nutritional care are improved knowledge and awareness of (the causes of) malnutrition to be able to recognize and to adequately act on possible signs of malnutrition.

*Causes of malnutrition* was identified as an important theme. Causes of malnutrition include physical and mental decline, mainly caused by ageing itself. This is not a novel finding. The decline in the ability to taste and smell, decreased appetite, effects of medication, metabolic changes and many other problems are known to influence food intake and uptake at an older age.^18^, ^19^ Additionally, social aspects such as small social networks, loneliness, absence of external stimuli and financial issues are known causes for malnutrition among OA.^20^, ^21^ Hence, a number of respondents, both OA and professionals, suggested to create more social and medical facilities in order to solve many of these social problems. In sharp contrast, the interviews also revealed that a large number of facilities already exist, but that OA and health‐care professionals are mostly unaware of these. Solutions might therefore not lie into organizing new facilities, but into promoting existing facilities and making sure they are known among OA and health‐care professionals.

*Knowledge and awareness* towards malnutrition in OA was the second theme that arose, and has been found to be low among both OA and caregivers and professionals. This could undermine timely recognition and treatment. These findings are in line with a previous studies.^13^, ^22^, ^23^, ^24^, ^25^ Even though several respondents gave suggestions how to inform and educate all stakeholders involved, the findings of this study do not yet provide specific guidance to this education.

The third theme appointed was *recognition and diagnosis of malnutrition*. This theme strongly relates to *knowledge and awareness*, but also to *communication between professionals*. Knowing how to recognize and diagnose malnutrition in an OA requires knowledge and awareness. A recent study has shown that many European educational institutions for nurses and GPs do not include malnutrition topics in their curricula.^23^, ^26^ Including the topic of malnutrition to the curricula of nursing schools and GP vocational training could be an important step in improving knowledge and awareness of malnutrition.^26^, ^27^ Additionally, welfare professionals indicated that they were mostly unaware which signs could indicate (risk of) malnutrition. Welfare professionals are frequently involved when OA have financial or social problems, and educating them may be helpful to improve identification of OA at risk of malnutrition. This could be done through initial curriculae or through in‐service training on this topic, preferably addressing a multi‐professional group (eg a community nurse, welfare professional and GP from a municipality). Additionally, professionals, but also caregivers, should be aware of the steps that need to be undertaken when a malnourished OA has been identified. They need to know which professional to contact in case malnutrition is suspected. This strongly relates to the following theme, *communication*.

Communication was a frequently discussed topic during the focus group interviews, both *between health‐care professionals*, *with OA and their caregivers*, and issuing the *transfer report*. As stated before, when (nutritional) problems are suspected by one professional, it is crucial that is communicated with other professionals. In short, in addition to knowledge about recognition of (signs of) malnutrition, communication with other professionals is considered equally important. However, efficient communication and information management is problematic^28^: offices or workplaces are often not situated in the same location, and organizing meetings with all professionals involved is time‐consuming and often logistically impossible. Therefore, innovative solutions should be sought, possibly using modern communication technology (ie through mobile phones and digital assistants, electronic group pages and worksheets),^29^ keeping the GDPR^30^ in mind.

Regarding *communication with OA and their caregivers*, the results of this study showed that OA themselves often feel ignored by professionals. This is in line with previous studies,^28^, ^31^ showing that patients are often not involved in the decision making of their treatment, while involving the patient in for instance discharge management showed positive results on several patient outcomes.^32^ Health‐care professionals should learn how to involve patients in the decision making regarding (nutritional) care, in order to make them feel involved in their own care process. This requires new competences, in approaching the client as a person, not as his disease.

Lastly, the *transfer report* was a frequently discussed topic. In many cases, transfer reports were delayed or never reached the proposed recipient. Additionally, nutritional information was often not included in the report. It is suggested that such reports should be issued in all cases and that there should be a fixed format, including nutrition(al problems) and possible steps undertaken and to be taken. A previous study investigated the effectiveness of transitional care and already concluded that, in order to reduce short‐term readmissions, transitional care should consist of communication between the primary care provider and the hospital.^33^, ^34^

Nutrition is one of the most important aspects in life. Besides the physical need of nutrition, social aspects play a major role. Herewith, nutrition is a topic that appeals to everyone. Contrarily, our study shows that no specific professional feels responsible for coordinating nutritional care. This is said to be caused by a lack of knowledge and/or It was suggested that besides the OA, one specific, or even several professionals should be (made) responsible. These results are in line with a previous qualitative study among 22 Dutch nutrition health‐care professionals,^13^ which concluded that awareness towards malnutrition was limited and it was unclear which professionals are responsible and which monitoring procedures are preferred. Thus, in order to prevent (further physical decline caused by) malnutrition in the elderly, a coordinator should be appointed. Depending on OA’s situation, the GP^23^ or nurse practitioner were suggested as coordinators, unless a home care nurse was involved. He/she was then suggested as the coordinator.

When discussing nutritional care across the continuum of health‐care settings, food preparation and supply is a topic that arises naturally. Many OA pointed out that food prepared and served in institutions is not what they are used to consume: variation is limited and OA cannot always continue their eating habits during admission. This topic was regarded as very important by OA and their caregivers.

One of the regions included in the interviews is a multicultural neighbourhood. Therefore, it was found important to also include OA with a non‐Dutch origin. Unfortunately, due to limited time and resources, only one couple with a non‐Dutch origin was included in the study, and no data saturation has occurred. However, the interview did reveal important results. Namely, the couple from Moroccan origin identified the language barrier and cultural differences (eg social relations, eating habits) as two of the largest barriers. As a result, they experienced that they were not always included in the decision making of their treatment. These findings are in line with a previous study which already showed that language and cultural barriers are severe barriers to putting shared decision making into practice.^35^ The results of this study imply that physicians should improve their skills to recognize the limitations within the communication with immigrant patients as well as improving the skills to acknowledge the barriers, which may help to ameliorate shared decision making in an intercultural setting.

With the increasing number of OA in the Netherlands, Dutch health‐care policy is aimed at encouraging OA to stay at home as long as possible. Herewith, vitality and independence are of great importance. Nutrition plays a large role to support this. When health problems arise and OA are compelled to be admitted to hospital or rehabilitation centre, nutritional care should be one of the most important topics in the communication and collaboration of professionals over the continuum of care. This study indicates that this is not yet the case.

Even though this study is one of the first in addressing this topic, a number of limitations have to be acknowledged. The name of the project, ‘Nutrition Passport’, was communicated as such to all respondents. This might have biased the respondents’ answers and steered them into the direction of a passport. Furthermore, the number of caregivers that participated in the focus group interviews was low, with only five respondents. It is therefore debatable whether the view of caregivers has been sufficiently clarified. Research has shown that caregivers could have an important role in nutritional care.^36^ Therefore, it might be interesting to further study the view of caregivers regarding nutritional care. Additionally, GPs were appointed as possible coordinators of nutritional care. However, only a small number of GPs participated in the focus group interviews. As a result, their opinion may not have been sufficiently taken into account. Lastly, one interview with a couple with a non‐Dutch origin took place. Future research is needed in order to outline the view and experiences of OA with a non‐Dutch origin.

Collaboration and communication with regard to nutritional care across the continuum involves many stakeholders. This study has clearly shown that collaboration and communication between different stakeholders is not yet optimal. Improvement could mainly lie in increasing knowledge and awareness regarding recognition and treatment of (mal)nutrition of professionals and OA themselves and their caregivers. Regarding communication, a solution is needed, keeping in mind the GDPR.^30^ Linking (digital) communication systems would be a solution that is embraced by all. Additionally, OA should take responsibility for their own health care and a professional should coordinate nutritional care, for instance the GP, the nurse practitioner or a home care nurse. Lastly, further research is needed to fully study the view of elderly immigrants.

The results of this qualitative study will be used to improve communication and collaboration of nutritional care across the continuum of health‐care settings, by developing a personal nutrition passport. Furthermore, suggestions will be made to improve transfer reports, including at least a patient’s disease, recovery, medication use, nutritional status, use of oral nutritional supplements and involvement of other professionals and timely availability of the report.^37^, ^38^

In summary, this study identified barriers and facilitators towards communication and collaboration of nutritional care across the continuum of health‐care settings. Many stakeholders are involved in the nutritional care of OA. Suggestions are given for improvement, such as nutritional education, improving possibilities for social coherence and establishing one coordinator of nutritional care.

## CONFLICT OF INTEREST

None of the authors declare a conflict of interest.

## Data Availability

The data that support the findings of this study are available on request from the corresponding author. The data are not publicly available for privacy or ethical restrictions.
